# Palliative care in rural areas – collaboration between district nurses and doctors: an interview study

**DOI:** 10.1186/s12904-023-01190-9

**Published:** 2023-06-15

**Authors:** Ulla Näppä, Elin Nässén, Idun Byberg

**Affiliations:** 1 Department of Health Sciences, Mid Sweden University, Östersund, Sweden; 2 Department of Surgery, Östersund Hospital, Östersund, Sweden

**Keywords:** District nurse, Home care, Interprofessional collaboration, Interview, Palliative care, Sparsely populated rural area

## Abstract

**Background:**

Palliative care requires major nursing interventions as well as medical interventions; thus, both district nurses and doctors are vital to the palliative team. Sparsely populated rural areas are characterised by large geographic distances with the nurses and doctors located far away from each other. If collaboration does not work, this can create challenges for district nurses when managing patients’ symptoms. The aim of this study was to describe district nurses’ experiences of collaborating with doctors-in-charge during palliative home care in sparsely populated rural areas.

**Method:**

Semi-structured interviews were conducted with 10 district nurses. Inductive content analysis was used to analyse the data.

**Results:**

The experiences of the district nurses are described under the overarching theme of *Experiences of acting as the patient’s advocate*, which is divided into two categories: *Feeling secure in oneself and the other person* and *Feeling alone when collaboration breaks down.*

**Conclusion:**

Consensus and coherence, or lack thereof, between district nurses and doctors affect how collaboration is experienced. Positive experiences are generated when the district nurse and the doctor share a holistic approach, while collaboration is experienced as dysfunctional when the doctor’s decisions are not consistent with what the nurse judges to be beneficial to the patient. An understanding of how collaboration across long distances is experienced in rural areas is necessary to enhance collaboration.

## Background

The objective of palliative care is to provide high-quality care in the final stages of life, which requires interprofessional collaboration between multiple actors such as nurses, doctors and social workers [1, 2]. Sparsely populated rural areas entail large geographic distances between these actors – a major problem when it comes to collaboration. Collaboration is perceived to be necessary, and when conversations between different professionals, such as district nurses and general practitioners, occur ad hoc or as scheduled meetings, threats to the safe, collaborative care of patients and treatment goals do not arise [2]. An understanding of how collaboration is experienced is essential in order to enhance collaboration and, thereby, the quality of the care provided.

The phenomenon of home care can be described in variable terms, usually based on the content of the care (e.g. in-home preventive assessment, palliative home care or home health care) or the objectives of the care, (home-care rehabilitation or hospital at home) or the form of service (medical care or home nursing care services) [3]. This study uses the term ‘home care’ in the setting of palliative needs. In Sweden, home health care is the responsibility of the municipalities and primary care, and hospital care is the responsibility of the regions. Collaboration between regional care services and home care is needed, as municipalities are not allowed to employ doctors [4]. Palliative care is divided into general palliative care and specialised multi-professional palliative care teams with specialist competencies. Patients with palliative needs living at home can be provided with palliative care from both general and specialised care givers [5]. The role of district nurse in Sweden is a specialist competence with extended education in nursing that includes palliative care, medical care, public health, and caring pedagogy. In home care and primary care, district nurses have the closest contact with the patients [6]. The role of general practitioner is a specialist competence characterised by the treatment of persons of all ages with all kinds of health-related problems. The work is generally done at primary care; palliative care is a part of the education [7]. In Sweden, of the estimated 90,000 people that die every year, 75,000 people receive palliative care [8]. Given today’s longer life expectancies and that more people are expected to die from chronic illness in the future, it is likely that more palliative care will be required in the future than the present day [9].

In Sweden, sparsely populated rural areas are defined as municipalities with a population of fewer than five individuals per square kilometre [10]. For residents in sparsely populated areas, the home is most often the preferred place for palliative care at the end of life compared to urban preferences. Reasons for this are the possibility of being surrounded by family and friends, and for indigenous people to be connected to land and family [11]. In rural areas, many elderly people tend to not want to leave their environment for as they have lived their entire lives in the same place [12]. This can be linked to the desire to conserve one’s integrity [13]. A home has a meaning such as a place, the meaning can also be a relation to the place or experiences of a place [14]. Home care refers to healthcare or medical care provided in the patient’s regular home or nursing home, where responsibility for the medical interventions is sustained over time, according to a documented care plan [4, 15]. As it optimizes quality of life, home care is considered to be a more effective type of care than hospital care [3]. Being cared for at home can help a person maintain a sense of self until the end of their life. To be at home gives the person a feeling of freedom and the possibility to remain active and alert and be involved in decision-making regarding their own care [3, 16]. Furthermore, it can entail the possibility of continuing to live a familiar and meaningful life. Although the choice to remain living at home in sparsely populated rural areas can mean long waiting times for medical assistance in the event of illness, the advantages of remaining in one’s home are deemed to outweigh the disadvantages by both primary care health professionals including district nurses and doctors [2], and patients living in rural settings [17]. A person’s choice of place for end-of-life care, just like their choice of place to die, is influenced by many factors, for instance, personal, relational and contextual circumstances [18].

In sparsely populated rural areas, district nurses face the challenges of large catchment areas, restricted access to support from other carers and safeguarding the anonymity of the ill patient in small communities, where most residents know each other and recognize the nurse’s house call by seeing the nurse’s car [19]. Healthcare, including advanced medical care at the end of life, is conducted in the patient’s private home, where the clinical eye of the district nurse is essential for controlling and monitoring symptoms [15]. In these conditions, a holistic view of nursing is needed, in agreement with Levine: advocating for each patient being seen as unique in contrast to the biomedical model, which reduces the patient to an illness, is necessary in palliative care [20]. All care should aim to conserve the unique individual, formed by their physical environment and psychological impressions from birth to death [21].

Interprofessional teamwork between nurses and doctors is necessary in rural home care where role clarity is important [22]. Few professionals work as close to one another as nurses and doctors; however, they are often portrayed as being each other’s opposites. Over time, relations between these professionals have changed and become more equal. Nevertheless, doctors still retain more power in decision-making. Collaboration between the nursing profession and the medical profession can be described as consisting of mutual respect and unequal power [23]. The interaction and communication between the two are fundamental for collaboration in palliative care, not least when the patient receives palliative care along with other services [24]. A cooperative relationship comprises mutual respect for each other’s professional roles and abilities. Frequent collaboration is essential to achieve the defined goals of the patient’s care [25], and district nurses and doctors need to collaborate to deliver good palliative home care. The nurse is considered an organiser and a link between the patient and doctors [2]. However, district nurses face collaboration-related challenges in terms of lack of time, limited access to patient files and difficulties in reaching doctors for prescriptions and consultation [22]. District nurses describe managing symptoms (e.g. pain), as the most challenging aspect of palliative care. However, the doctor’s task is to manage the patient’s psychological and physical symptoms by aiming for the greatest possible symptom relief [26]. In Sweden, when a person with palliative needs deteriorates, the treating doctor is obliged to have a talk about the disease’s breakpoint with the patient, in Sweden referred to as the *breakpoint conversation*. The breakpoint conversation should consist of information about diagnosis, possible treatments and the predicted course of the disease. A nurse is always supposed to be attendant [27]. Previous research demonstrates that palliative care requires major nursing interventions as well as medical interventions [26, 28–30]. Sparsely populated rural areas are characterised by large geographic distances and can entail long distances between the locations of different professionals. Nevertheless, people in rural areas prefer home care, even if it means that they must wait for a long time to get help when needed. This can pose a challenge to collaboration between district nurses and doctors.

Currently, research into how collaboration is experienced in palliative home care in rural areas is lacking.

## Method

The aim of this study was to describe district nurses’ experiences of collaborating with the doctor-in-charge in palliative home care in sparsely populated rural areas and generate greater understanding of the importance of properly functioning interprofessional collaboration. A qualitative interview study design was used to explore the experiences of district nurses, which was analysed using inductive content analysis. According to Polit and Beck [31], qualitative interviews tend to be holistic and aim to achieve an understanding of the whole.

### Participants and procedure

District nurses working in five sparsely populated rural municipalities in Northern Sweden were invited to participate in the study. In all the municipalities palliative home care was provided in collaboration with the general practitioner, on-call doctors and/or specialist doctors in palliative care. In this study, the general practitioner and the specialist doctor in palliative care collaborated on patients with palliative care needs if there had been a referral to the specialist care team. The inclusion criteria for participants in this study were district nurses with experience of working within palliative home care in sparsely populated rural areas. Convenience sampling, sometimes called volunteer sampling, to find participants in rural areas was conducted by contacting heads of municipal home care and informing them about the study verbally and by sending out emails that contained information on the study and a written consent form. The heads asked their district nurses whether they were interested in participation, and then forwarded the interested nurses’ contact information to the researchers. The participants were selected for inclusion due to meeting the inclusion criteria and being interested in discussing the research subject [32]. Two men and eight women participated in the study. For further demographics, see Table 1.

**Table 1:** Demographics of participants (N = 10)

		Md/Mean
Age	35–65 years.	56.5 (51.6)
Time working as DN	2.5–40 years.	10.5 (14)
Work schedule	Mainly night shift: 2 Day shift: 8	

At the time of the interviews, all participants were employed within home care. The participants were responsible for between 17 and 38 home care patients. During daytime work on weekdays, the catchment area could be 170 km in one direction. During night shifts and weekends, the nurses were responsible for all residents covered by home care within an entire municipality, which could involve distances of more than 250 km one way.

Semi-structured interviews were the chosen method for data collection. The order of the questions was fixed, however there was a possibility for the participants to speak more freely, as if structured questions following the form of a questionnaire had been posed. Semi-structured questions are also appropriate for research into views and opinions and allows for more in-depth answers and questions [31, 33]. The interview questions for this study were developed by the authors for this study. A pilot test of the interview was carried out with a registered nurse working in a hospital ward with experience in home health care. The aim of this was to test the interview questions. After the pilot interview was conducted, three optional follow-up questions were added to the interview guide. The interview guide consisted of five demographic questions, two opening questions, five probing questions and one closing question (see Table 2).

**Table 2:** Interview guide

Demographic question	How old are you? For how long have you worked as a district nurse? For how long have you worked at this workplace? For how many patients are your responsible? How big is your catchment area in km^2^?
Opening questions	Can you tell us about a situation when the collaboration between you and the treating doctor worked well in palliative home care? Can you tell us about a situation when the collaboration between you and the treating doctor did not work well in palliative home care?
Probing questions	Can you tell me more? Could you please clarify that? What feelings arouse at that moment? How did you act in the situation? Have you any further demands?
Closing question	Is there anything else you would like to address?

Open-ended probing questions were used to draw out deeper answers and generate further reflections.

The interviews took place during May and June of 2019 and were recorded using digital audio-recording equipment. They lasted between 18 and 51 min (Median = 38 min, Mean = 37 min). The participants were invited to choose the time and place for their interviews and were given the choice of a telephone or in-person interview out of consideration for the possibility of the long distances that may affect their participation. This resulted in five in-person interviews, three telephone interviews, and one joint interview with two district nurses, who specially asked to be being interviewed together. All the interviews were conducted by the second and third authors together, as a part of a master’s exam; they had received theoretical and practical training during education. The interview procedure was that the third author asked the standardised questions and took a more passive stance, listening and observing, while the second author was more active, asking follow-up questions to elicit deeper explanations and taking notes. According to Monforte and Úbeda-Colomer [34] there are very few methodological papers exploring this method of data collection. The interviews took place with the third author reading out the aim of the study and thereafter asking the demographic questions, two opening questions and one closing question from the interview guide. During the interviews, the second author asked additional follow-up questions to stimulate further reasoning. During the interview with the eighth district nurse, no new insights emerged compared to the prior interviews. To be sure no data was missing another two interviews were carried out to ensure information power. As the new data collected tended to be redundant information power was deemed to have been reached. According to Malterud, Siersma & Guassora [35], the concept of information power in the sample is the most important determinant of the number of interviews. The greater the information power the interviews hold, the lower the number of interviews is needed. The aim, selection criteria, theoretical association, quality of dialogue and analysis method determine the number of participants. They postulate that homogeneous groups require fewer participants than when a more heterogeneous group is studied. In this study, the participants were homogeneous based on profession, geography, workplace, and education, but they were relatively heterogeneous in terms of age, gender and number of years in the profession. These criteria fulfil the requirements for information power [35].

**Fig. 1:**
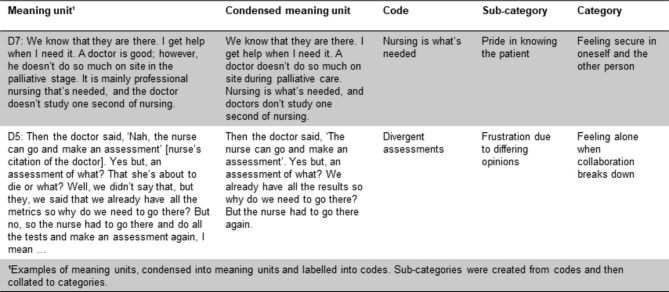
Examples of the analysis process

The interviews were transcribed verbatim by the second and third author and analysed by all three authors using qualitative content analysis, in conformance with the procedure described by Graneheim and Lundman [36]. The interviews were read several times separately to gain an overall picture and understanding of the research topic, paying attention to latent content including irony, laughter, intonation and similar to capture the implied meaning of the interviews. The interviews were analysed impartially by all the authors using an inductive approach, dividing the text into meaning units in a joint document. Meaning units that fitted the aim were selected jointly. These were then condensed without loss of the content and coded into labels. The codes were reviewed independently and situated within the context of the entire transcription to ensure that their content had been reflected correctly. All the codes were re-read and discussed by the authors to identify the codes that illustrated the same topic. Then, they were collated into categories. Sub-categories were created from the codes to provide a comprehensible view of the similarities and differences that emerged from the content analysis (see Fig. 1 for examples). To support the analysis process Erlingsson and Brysiewicz’s [37] description of the practical application of the method was also used. The three authors met for several reflexive discussions during the analysis process, going back and forth in the analysis to reach a consensus to defend the data’s credibility (cf. Graneheim & Lundman [36]). All researchers had some level of prior understanding of the research topic through work placements; therefore, they continuously strived for neutrality by setting their preunderstanding aside to avoid impacting the results.

Initially, a manifest analysis was performed; however, this was not considered to reflect the sentiment of the interviews as the authors critically found that the categorisation did not mirror the inner feelings or implied meanings of what the district nurses shared in the interviews. Therefore, the analysis was carried out jointly by the authors with greater consideration given to the latent content, which was regarded to better reflect the stories of the participants. Once a clear picture of the content had been attained, an overarching theme was defined. The translation from Swedish to English was carried out during the categorisation and re-evaluation of the concepts.

## Results

**Fig. 2:**
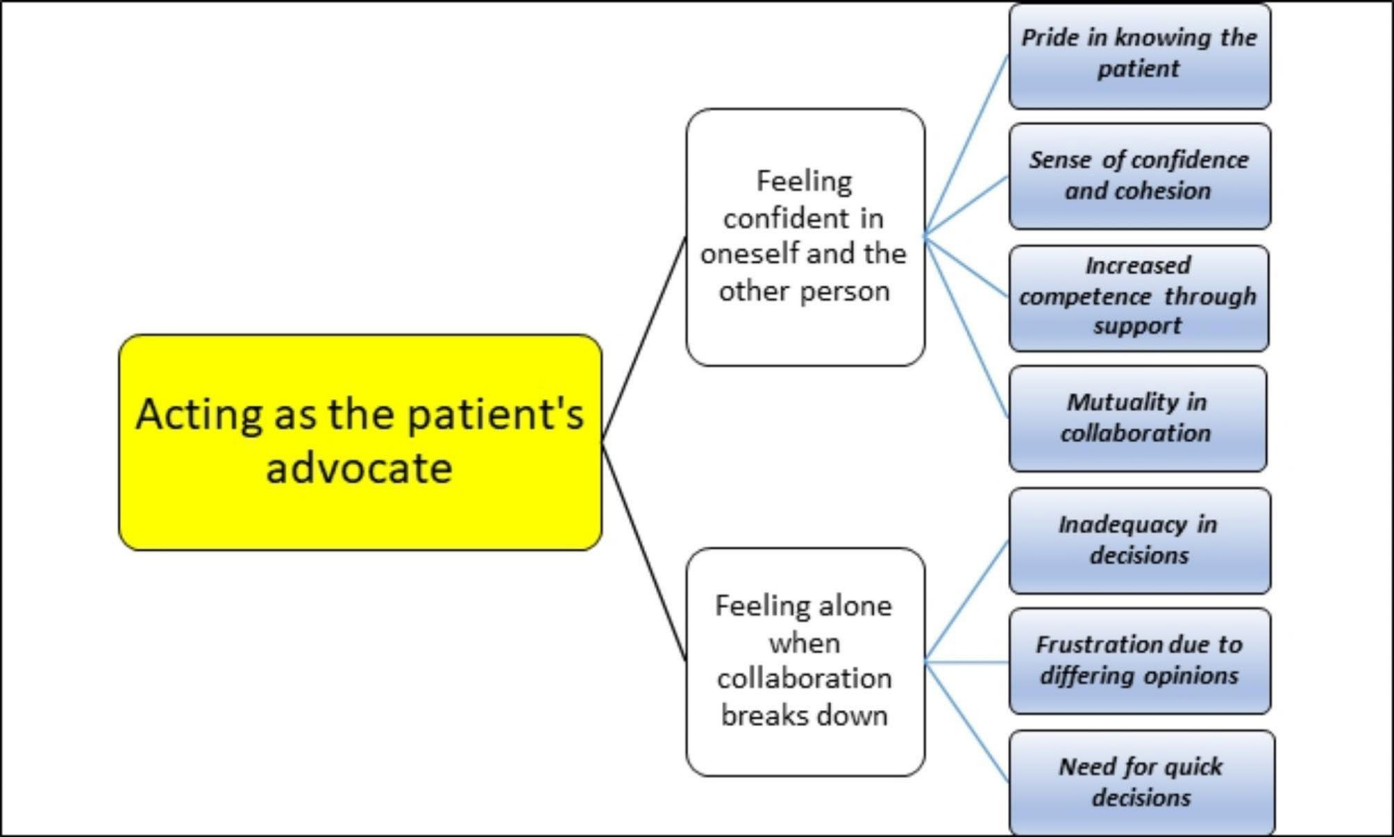
The theme, categories, and sub-categories

The analysis of the interviews with 10 district nurses regarding their experiences of collaborating with doctors resulted in one theme, two categories and seven sub-categories. The overarching theme *Acting as the patient’s advocate* represents the underlying meaning of the interviews. Irrespective of the dissimilarities between the district nurses in terms of length of professional experience, gender and work schedule, collaboration with doctors was reflected in a similar manner. As the people responsible for the patients’ care, the district nurses saw themselves as being closest to the patients and as the patients’ advocates during collaboration with doctors, which revealed both properly functioning and dysfunctional experiences in one and the same meeting. In the functional meetings, the district nurses felt confident in themselves and the other person, and in the dysfunctional meetings the district nurses felt alone and that the collaboration broke down (see Fig. 2).

Collaboration with general practitioners, on-call doctors, and/or specialist palliative care doctors was investigated in the interviews. When discussing any doctors in this section, they have been labelled as ‘doctor’.

### Feeling confident in oneself and the other person

The district nurses shouldered great responsibility in palliative home care, which they described as having plenty of power in making major decisions. The general practitioner would turn to the district nurse to find out the patient’s wishes, which the district nurse usually had knowledge of. The district nurse’s role was likened to that of a co-ordinator, linking together different care contacts. The nurses thought they knew the patients better than any other carer, largely due to building relationships with them over a longer period, even before the palliative phase. The district nurses felt they had the great responsibility of advocacy on behalf of the patient and conveying issues as needed. The district nurses’ experience of palliative care gave them confidence and security, and it confirmed their feeling of being correct in their assessments. The greater their experience, the better their relationship with the treating doctor and the easier it was to collaborate. Furthermore, it emerged that the experience of having worked together for a long time facilitated collaboration and reaching consensuses. D6 explained it in this way:

‘When I was new, then there was nothing; I didn’t know them and they didn’t know who I was, then everything was much more difficult. Now that I know the patients and I know the doctors and they know who I am, then it’s easier.’ (D6)

Contact with on-call doctors, who had varied experience of palliative care and were located at a long distance from the patients without having met them, required the district nurses to stand up for what they believed to be right. Often, the district nurses had a plan when the doctor was contacted; thus, they would suggest what had to be done. For the most part, the on-call doctor accepted such suggestions. The district nurses often initiated discussions about a different level of care, such as a transition to palliative care. Good knowledge of the patients and being able to monitor the course of the care enabled the identification of gradual deterioration. When initiating a change in the care, the district nurses used strategies in discussions to reach consensuses with the general practitioners; thus, their views were often well received by the doctors. When they did not receive backing, their strategy was to contact another doctor. The district nurses were often the ones who knew the need and purpose of the general practitioners’ house call and what was expected to be done. When possible, the district nurses strategically chose the general practitioner that they knew from experience was willing to do house calls. One participant described it as follows: ‘If I want a house call, I choose the right doctor […] You can become sneaky as a district nurse.’ (D7).

The district nurses had a desire for proactive planning, which was strongly linked to professional pride. It was grounded in a desire to act efficiently in the event of unexpected deterioration without the need for urgent contact with the doctor. They did not want to have to call the on-call doctor in the middle of the night due to a lack of prescriptions. District nurse D7 described that as follows: ‘A doctor is good; however, he doesn’t do so much on site in the palliative stage. It is mainly professional nursing that’s needed, and the doctor doesn’t study one second of nursing’ (D7). They appreciated being able to make their own decisions, however, as D8 put it, ‘[I] know where the limit up to the doctor level is’ (D8) and knew when the doctor’s involvement was needed. Breakpoint conversations with the doctor were not always considered necessary as the district nurses and patients had already managed it in their own way; it did not need to be more complicated than that. As explained by D6:

‘What you could say is that even though many [patients] don’t get to have a breakpoint conversation, there might not actually be a need for one. Because, if I’ve known the patient for a long time … well, we’ve managed it. … And it feels like that’s sufficient. Somehow you know the patient so well that we’re already there. So, you could say that, unofficially, we’ve already had that conversation because we know them.’ (D6).

#### Sense of confidence and cohesion

The district nurses preferred proactive planning, even if this meant that decisions needed to be revised, as the process of dying could take a long time. Having everything prepared when the patients deteriorated was described as giving a sense of confidence, since the district nurses had the tools to help. At the end of life, contact with the doctor was rarely needed because the breakpoint conversation had been held, the medicine prescribed and the declaration of death delegated to the district nurse. Most district nurses provided for all eventualities, and a sense of confidence emerged when plans for all imaginable scenarios were in place. D8 explained it as being one step ahead:

‘You must be one step ahead in palliative care. You must never be surprised; you must consider the idea that an unpredictable scenario can happen. It’s good to be prepared and ready for it. Especially out here in the outback where nurses’ shifts end at 5pm.’ (D8).

The district nurses related the experience that nowadays, they carried out more medical duties than in the past, when the general practitioner always visited the patient. Previously, general practitioners issued the declaration of death or removed a pacemaker after the patient died. The organisational structure within which doctors work today has resulted in such medical duties being delegated to district nurses more often. However, the district nurses did not mind performing these tasks. They stated that these medical duties felt natural since they had had the closest contact with the patients. Nevertheless, this required good planning so the district nurses could feel confident in performing the tasks. D3 explained when it worked well:

‘…when the death is expected, where there is… where the doctor or nurse have had a breakpoint conversation, then they [the doctors] have delegated declaration of death to us district nurses: Then they don’t declare deaths, it’s us who do it […] because it is still I who have had contact with the patient, close contact, in the last months, weeks or days of life and I know the relatives and staff, so I have nothing against performing that task, really.’ (D3)

A good plan included asking whether a house call for the doctor’s breakpoint conversation with the patient and significant others was included. The district nurses argued that the specialist doctors in the palliative care team were considered to be particularly skilled at these conversations. They explained in a straightforward manner to the patients, significant others and district nurses that the end of life was close.

In most cases, the district nurses and doctors agreed on the level of care, prognosis and treatment, which gave the district nurses a sense of confidence and structure. With patient consent, authorised care personnel could access information in medical files entered by hospital care providers. This access helped the district nurses understand the doctor’s reasoning for the treatment. However, the national patient register was described as functioning only as one-way communication, since the district nurses knew doctors could not read the district nurses’ documentation. They assumed two-way communication would lead to a better overall view of the patients’ care. District nurses also expressed a wish for digital technology and suggested conversations via video-link with the patient and/or the doctor, arguing that it would widely improve collaboration and increase the involvement of the patients. 

#### Increased competence through support

The specialist doctors in the palliative care team served as an additional source of advice with consulting the general practitioner or the on-call doctor. They could also enact faster decisions. The specialist doctors in the palliative care team were better able to support district nurses and patients than general practitioners as their schedules gave them more time to listen in emotionally strained times. This supported the district nurses in dealing with the patients’ and their significant others’ grief and understanding that what they had done was good and enough.

‘They [specialist doctors] said that if I handle things that are tough, for example a younger person with children … then they’ll come and we can talk […] I mean… you also have to learn that it’s not me… it’s not my grief, it’s not mine, instead, you have to understand that you’ve done all that you can […] a general practitioner never does that… I mean…’ (D2).

The support and experience the district nurses received through collaboration with the specialist doctor in palliative care increased the district nurses’ competence in palliative care. The specialist doctor in the palliative care team also provided valuable support thanks to their thorough knowledge of the patients. The district nurses wished that this approach in palliative care could be adopted and transferred to the collaboration with the general practitioner. However, general practitioners who had long-term experience in the same workplace also gave the district nurses similar support. They had good knowledge of the patients and knowledge of sparsely populated rural areas, which made decision-making easier. D8 described it as follows:

‘Our two general practitioners are aware of what it’s like to live out here in the rural areas, what struggles and distances we have. Many elderlies and many elderly old persons live here. They understand the situation in a completely different way than an on call-doctor who might come from urban Sweden. Now, I call the doctor and say the patient’s name and that he is having difficulties breathing and moving. The patient lives alone in his own apartment. The doctor knows immediately who I am talking about and can see the picture in front of him. The doctor immediately understands that now it is unmanageable and it’s easier for him to plan.’ (D8)

Good collaboration with the general practitioners was a contributing factor to the district nurses choosing to remain at the same workplace.

#### Mutuality in collaboration

The district nurses’ central role in palliative home care was to give confidence to the patients and their significant others, and the doctors had a lot of trust in the assessments made by the district nurses. The district nurses perceived themselves as the doctors’ eyes and ears when caring for patients in palliative care, and as such, verbal gratitude from the general practitioners was appreciated. An example of mutuality was described by D8:

‘You can understand that the doctors are dependent on us, we make assessments, and they trust in our experience. That’s the way it is, both Dr. N.N.1 and Dr. N.N.2 say: “You are valuable, you know, I mean, without you this just wouldn’t always work.” And that’s how it feels sometimes too… there’s give and take, and you feel yourself grow.’ (D8)

Several of the participants described special situations when they appreciated that the general practitioner had gone the extra mile, for example, when the general practitioner could not fit a house call into their working day, and instead did it on the way home from work. Another example was when the district nurse did not have the time to perform a lengthy home treatment, so the general practitioner did it themself to save the patient a journey to the hospital.

### Feeling alone when collaboration breaks down

#### Inadequacy in decisions

Being alone without a plan for a deteriorating patient and with an unavailable doctor was experienced as dysfunctional collaboration. When a patient was dying and contact with the doctor took place solely by telephone, the district nurses felt that they were left to their own judgements and that the palliative care given was based on the phone conversation.

‘…when you don’t have adequate prescriptions, then you end up a bit helpless. You feel that you can’t relieve symptoms, and so on… Then, you don’t feel satisfied. You know it could have been done in a better way if it had been prepared so that you could have given the medicine that was needed.’ (D10).

As nobody questioned the district nurses’ judgement, it made them wonder whether anything could have been done differently. Sometimes, the district nurses felt that they had caused the patient’s death.

The district nurses’ judgements were primarily based on the patients’ care needs from the perspective of nursing, while the doctor made judgements from a medical perspective. House calls from the general practitioners were requested to better optimise the patient’s medical treatment. D5 explained the medical perspective as observing ‘other things’: ‘I see the patient from my [nursing] perspective, need of care and well-being like that, but if the doctor comes out, he or she can see… they see other things, as well…’ (D5).

However, it was often the district nurses who conveyed medical decisions to the patients. Contact with the general practitioner or on-call doctor was prompted when the prescribed pain relief was not considered sufficient and the district nurse was uncertain about whether more medication could be administered. The district nurses assumed that the rapid development of advanced technological medical aids could lead to more patients being given palliative care at home, which in turn would increase the demand for good collaboration between district nurses and doctors. General practitioners or on-call doctors were regarded as being more cautious and parsimonious and less prone to adjust the prescribed dosage for the patient compared with the specialist doctors in the palliative care team. General practitioners and on-call doctors were also less prone to prescribe medicines that required advanced technological medical equipment for administration. In most cases, the on-call doctor did not know the patient and could therefore be uncertain about whether the end of the patient’s life had been reached. When the patient deteriorated unexpectedly and documentation from the general practitioner was missing and the district nurse needed a decision about limitation of care, the district nurses perceived the on-call doctor as being afraid to make decisions. D5 described the situation as follows:

‘They [on-call doctors] are a little afraid, they don’t want to do something like this, what’s it called, breakpoint conversation. They don’t want to set zero CPR (cardiopulmonary resuscitation) and they don’t want to give prescriptions either, so if things like this happen at on-call time the on-call doctor can be, a bit annoyed, and then maybe they don’t want to… they try to leave it until the next day instead…’ (D5).

It was even more difficult if the district nurse on site was not the patient’s regular nurse and thus, did not know the patient. D1 said:

‘Because it’s not good, when the patient gets worse… in the weekend… the nurses don’t know the patient as well as I do… maybe there’s no delegation for declaring deaths… Because that happens when patients die very fast… and there was no delegated declaration, and no prescriptions…’ (D1).

#### Frustration due to differing opinions

Lack of consensus, that is, when the doctor was of another opinion, was frustrating for the district nurses. In the absence of a decision regarding limitation of care, the district nurses could end up in a situation where they needed to send deteriorating patients to hospital even though they considered it more ethical to provide good care in the patients’ homes. However, inadequate planning made this impossible. Scanty knowledge of the patient was problematic as it could mean that the doctor refused to make decisions on limitation of care, despite the district nurse’s knowledge. One such example was given regarding a 96-year-old patient with incurable cancer who no longer wanted to live:

‘There’s nothing that says the patient can’t go to the hospital. But we need a dialogue that should be consistent with what feels ethically good. It’s not so fun to start CPR on some lady who is 96 years old and doesn’t want to live but there has not been a decision [on zero CPR] made. Then you end up in a dilemma.’ (D2)

This placed the district nurses in an ethical dilemma as they would need to perform CPR in the event of cardiac arrest. District nurses assumed that when there was lack of consensus in such situations, it would have been easier if they could make decisions for themselves, rather than be reliant on the doctors. On some occasions, patients were subjected to pointless testing rather than being given relief because the doctors did not follow the district nurses’ input. It was considered frustrating when the patient had several treating doctors but none of them wanted to make decisions on foregoing life-sustaining care or what would be an ethical decision for the patient.

‘It can be diabetes, or it can be the kidneys, or it can be [anything]… they have cancer somewhere and there’s loads of doctors at the hospital thinking one thing and another, and the patient is just getting sicker and sicker and nobody’s seeing the picture.’ (D7)

There was an implicit expectation that certain things should be done prior to contacting the on-call doctor. However, there was a lack of written procedures describing what should be done, leading to frustrating situations. The district nurses described being reprimanded by the on-call doctors, for example, when asking for prescriptions based on reports from colleagues working the earlier shift, without having seen the patients themselves, or the on-call doctor suggesting that statements from the patients’ significant others could not be trusted. On-call doctors were sometimes irritated about being contacted at night for requests regarding treatments and stated that these should have been arranged during the day through more thorough planning. The situation was described by D5 as follows:

‘We are not allowed to give morphine without calling. And you don’t want to call the doctor at two in the morning if something like that happens. It’s the same with palliative care […] if things weren’t planned, we must call and wake them up. Some doctors get very upset when you call at night, like; why do you call me now at two o’clock about something like this? I mean they can say things like that… they can get really angry when you call about something they think is unnecessary…’ (D5)

Occasionally, the district nurses departed from the doctors’ decisions and made assessments based on their nursing perspective, in correspondence with the patients and their significant others, but which were not consistent with the assessment of the on-call doctors. This conduct gave the patients and their significant others a strange picture of the caring profession as the district nurses and the doctors pulled in different directions.

#### Need for quick decisions

The district nurses perceived it as negative when doctors were difficult to reach or unable to make quick decisions. Given the large geographic distances and long travel times, quick decisions were required in order to have time to organise and administer the prescriptions. The general practitioners and on-call doctors were described as more difficult to reach and slower in making decisions and issuing prescriptions than the specialist doctor in the palliative care team.

‘Because, if I’m going to have time to administer the prescriptions to help that patient… Travelling times are so long… This means that you can’t sit and wait for prescriptions forever… because you must have the time to do those things then, in a reasonable amount of time…’ (D9).

Time for doctors’ rounds was divided across several district nurses and was the only time allocated with the general practitioner. The rounds were generally carried out by the district nurses and the general practitioners in the absence of the patients at the doctor’s office at the health care centre based on the district nurses’ observations and conversations with the patients. Decisions were later explained to the patients by the district nurses. The district nurses often took blood samples without prior consent from the general practitioner to compensate for the shortage of doctors. The district nurses also had to ensure that the patients got house calls to which they were entitled from the general practitioner. House calls were expected to be done during the rounds and they were thus complicated to implement if the patients lived more than 20 km away. When the district nurses needed support from the general practitioner over and above the rounds, it impinged upon the general practitioner’s planned work at the healthcare centre. Shortage of time was not so obvious when the specialist doctor in the palliative care team was in charge. The specialist doctor in palliative care worked based on a different kind of planning and could spend three hours in a patient’s home, contrary to the 20–30 min the general practitioner had for a house call. The district nurses described that the amount of time spent by the specialist palliative care doctor on a house call gave an entirely different perspective to the care compared with what the general practitioner had to deal with:

‘That is, they [general practitioners] have a little amount of time… when you go on home visit with the palliative care team and the doctor is participating, then it can take one and a half, two or even three hours depending on what needs arise, the relatives are often involved… but we don’t have that kind of time perspective with any general practitioner… no…’ (D2)

## Discussion

The underlying meaning of the interviews lead to the overarching theme *Acting as the patient’s advocate.* The district nurses’ reflections about their collaboration with doctors was irrespective of the dissimilarities in terms of length of professional experience, gender and work schedule. Being responsible for the patients’ care, the district nurses saw themselves as being closest to the patients and as the patients’ advocates during collaboration with doctors. This revealed properly functioning collaboration experiences where the district nurses felt confident in themselves. However, dysfunctional collaboration experiences where the district nurses felt alone and that the collaboration broke down emerged. The results are discussed considering the overarching theme of the study.

The study results revealed several factors that could affect district nurses’ experiences of the collaboration with doctors during palliative home care in sparsely populated rural areas. Based on Levine’s [21] theory of nursing, the goal of nursing is to conserve the wholeness of the patient. The holistic approach of district nurses and their efforts to conserve the patient as a unique individual is likely to affect their perception of feeling confident in themself and the other person when collaborating with doctors, not least because the social relationship is interpersonal (cf. [21]). According to Snelgrove and Hughes [38], the social and emotional aspects of the patient are central to holistic care and are important complements to the biomedical perspective. The need for both nursing and medical knowledge becomes clear when the nurse feels alone and the collaboration breaks down due to different perspectives. This creates feelings of loneliness in making assessments of patients’ conditions. Similarly, research demonstrates that the absence of support from doctors negatively affects the patients’ dignity, thereby placing the district nurses in an ethical dilemma that gives rise to feelings of discomfort [30, 39].

The results show that prior experience of working with palliative patients gives district nurses confidence and security in their role, and a greater ability to support the patient in conserving their structural integrity, as also described by Levine [21]. The district nurses had a feeling of pride, strongly tied to thorough knowledge of the patient, which according to Snelgrove and Hughes [38], facilitates the prioritisation of decisions, and the determination of what is beneficial based on the patient’s needs. The district nurses in this study had the patients’ best interests from a holistic view in mind. These factors of caring are consistent with Levine [21] in that a holistic view is taken to make optimal decisions for the patients’ best interests.

Proactive planning gave the district nurses the confidence to work independently and satisfy all imaginable needs of the patients, which, according to Karlsson and Berggren [13], also gives the patients confidence. However, protection of the patients’ integrity and symptom relief, required collaboration with doctors. The results of this study demonstrate that the district nurses’ frustration arose when no decisions were taken regarding limitation of care, sometimes leading to the deterioration of the patients and their unnecessary admission into hospital. Research shows that proactive planning creates better chances for patients to stay at home when they deteriorate and thereby retaining their self-identity and social integrity [13, 40]. According to Levine’s [21] four principles for delivering care at home, proactive planning is preferred, as it conserves the wholeness of the individual. In contrast to the results of this study, other research shows that district nurses can feel uncertain despite having access to clear treatment plans; it can therefore be assumed that uncertainty varies depending on the individual [30].

There is a level of medical competence included in the profession of the district nurse. However, according to these results, the district nurses preferred for the doctor to first make a medical assessment within their area of responsibility so that the district nurses were given the tools to act freely within a defined scope. A continued hierarchical positioning, in line with what Price et al. [23] emphasise, is needed so that district nurses do not transgress the boundary of the doctor’s level. When the district nurses noticed that the patients deteriorated and suggested palliative care, it was often received well by the general practitioners when collaboration was good. Other research suggests that good relationships with the doctors are needed to facilitate collaboration, generate trust, and showing respect for the other profession [39, 41].

When contacting the doctor, the district nurses had a plan in mind based on their holistic view of the patient. So, if the doctor did not dare to base their decisions on the district nurses’ description, frustration arose. Corser points out that not trusting the district nurses’ assessment was perceived as disrespectful, and as pointed out, respect is a prerequisite to collaboration [25]. Other research shows that respect for the nursing profession can vary depending on culture, hierarchical traditions, and education. For example, Italian nurses perceived that their profession was disrespected by doctors who actively belittled them, as if they felt threatened by the growing status of nursing [42]. In contrast, English doctors demonstrated a high appreciation of the nursing profession [41]. However, nurses do not always understand the complexity that doctors face in decision-making regarding palliative care [43]. As such, frustration associated with interprofessional collaboration appears to be grounded in divergent perspectives. Increased understanding of each other’s area of responsibility could probably improve collaboration between the professions. However, not all general practitioners are positive in collaboration with district nurses, which could be a reflection of the awkwardness and discomfort they feel in their role as doctors today [44].

Most doctors had great confidence in the district nurses’ knowledge and how they kept them informed about the patient, which improves the patients’ chances of being able to stay at home if their conditions deteriorates, and thus, keeps the patient’s wholeness respectfully intact [45]. The specialist doctors within palliative care and general practitioners with good experience of sparsely populated rural areas provided good support to the district nurses as they had solid knowledge of the patients. Generally, general practitioners were seen to be more parsimonious in their prescriptions than the specialist doctors in the palliative care team. However, Levine stated that described that every patient should be regarded as a unique individual; thus, prescriptions probably need to be tailored [21]. Based on Levine’s theory of nursing [21], the perceived lack of a holistic view from the general practitioners may be one reason for the district nurses to feeling that their collaboration is dysfunctional. While collaboration is affected by organisational factors such as education, it is also affected by personal attitudes and communication tendencies. The education of specialist doctors in palliative care teams includes more of a holistic approach; thus, they share the same perspective as district nurses, thereby making it easier to collaborate [25].

A need for quick decisions was considered a cause of dysfunction, as in other studies, district nurses [30] and general practitioners [46, 47] were found to believe that palliative care is time-consuming. Doctors’ lack of time can also be presumed to be the reason for district nurses becoming the patients’ advocates [48]. General practitioners consider that house calls an opportunity for doctors to gain a holistic view of the patient, which can help them to make medical decisions to benefit the patient [45, 46]. However, it was simultaneously pointed out that general practitioners do not have the time to perform house calls to the extent they would like to [46]. Thus, a holistic view and time can be considered as prerequisites for each other. In this study, the general practitioners and on-call doctors in rural areas lacked time to perform house calls to the extent the district nurses’ thought was needed. However, it has been shown that general practitioners in sparsely populated areas in Great Britain allocate more time to house calls and feel less stressed than those in densely populated areas [46]. In Norway doctors were more willing to go the extra mile for the patients’ best interests in rural areas [40]. It is possible that this is similar in sparsely populated rural areas in Sweden, and if so, a shortage of time would probably have been more evident if this study had been carried out in a city environment.

## Limitations and strengths

With regard to study limitations, the choice of method was essential to ensure the validity of the content analysis (cf. [36]) and an inductive analysis was appropriate for addressing the aim of the study when unexpected answers were given by the participants (cf. [49]). To ensure transferability, the context, sample, participant characteristics, data collection and analysis process were described as clearly as possible. Due to the particularity of the situation, the results of this study may not be generalisable. Interviews were conducted in three different ways to provide the participants with equal possibilities to participate due to long distances in rural areas [50]. As the interviewers were not particularly experienced, they conducted the interviews together. Therefore, the interview questions were standardized to compensate for variability in skills [51]. However, according to Monforte [34], this strengthens the data collection. In the joint interview, the participants may have developed each other’s thoughts, which enriched the data. The in-person interviews and telephone interviews revealed similar experiences; thus, the different interview methods were not seen to negatively affect the results. However, the pilot interview provided important knowledge on questioning. During the analysis, it emerged that alternative follow-up questions could have been beneficial for drawing out more comprehensive descriptions. All the authors contributed to the verification of neutrality as well as intersubjectivity by holding back preunderstanding and having joint discussions throughout the course of the research. Collaboration in coding increased comprehension, supported intersubjectivity and lead to judicious interpretation of the material [52].

## Conclusion

Due to the organisation of palliative home care in sparsely populated rural areas, the district nurse must be the eyes and ears of the doctor. The organisation also leads district nurses to act as the patients’ advocates in the collaboration with the doctors, ensuring the patients’ best interests based on a holistic view of nursing. District nurses may feel professional pride, confidence in their abilities and appreciation from the doctors if they also receive the support they believe they need. Shortcomings in the interprofessional collaboration give rise to frustration and a sense of inadequacy, and shortage of time makes collaboration harder. This study presented the experiences of interprofessional collaboration from the perspective of the nursing profession: the study only shows the nurses’ side of care. The perspective of doctors would have been valuable to balance the results. As the discussion shows that collaboration differs in different countries, a broader study from the perspective of both nurses and doctors should have been performed. It is important for decision-makers within palliative home care in sparsely populated rural areas to have this knowledge to increase understanding of the complexity of the provision of such care. Enhancement of collaboration also requires an understanding of the other profession’s experiences in the same context. The education of nurses’ and doctors’ needs to include the practice of collaboration e.g., students in interprofessional classes. Therefore, an equivalent analysis of doctors’ experiences of collaboration with district nurses during palliative home care in sparsely populated rural areas is suggested as a topic of future research.

## Data Availability

The datasets used and/or analysed during the current study are available from the corresponding author upon reasonable request.
