# Correction: Palliative care in rural areas – collaboration between district nurses and doctors: an interview study

**DOI:** 10.1186/s12904-026-02139-4

**Published:** 2026-05-25

**Authors:** Ulla Näppä, Elin Nässén, Idun Byberg

**Affiliations:** 1 Department of Health Sciences, Mid Sweden University, Östersund, Sweden; 2 Department of Surgery, Östersund Hospital, Östersund, Sweden

**Correction: BMC Palliat Care 22, 73 (2023)**

**https://doi.org/10.1186/s12904-023-01190-9**

Following publication of the original article [1], the author name, email address and Initials in the Authors’ contributions should be updated as follows:

Idun Winqvist“ should be changed to “Idun Byberg“.

The email address, idun.winqvist@miun.se, should be changed to: idun.byberg@miun.se.

The initials “IW” should be “IB”.

The original article has been updated.
